# PRMT5-mediated arginine methylation of TDP1 for the repair of topoisomerase I covalent complexes

**DOI:** 10.1093/nar/gky291

**Published:** 2018-04-30

**Authors:** Ishita Rehman, Suparna M Basu, Subhendu K Das, Sangheeta Bhattacharjee, Arijit Ghosh, Yves Pommier, Benu Brata Das

**Affiliations:** 1 Laboratory of Molecular Biology, Department of Biological Chemistry, Indian Association for the Cultivation of Science, 2A & B, Raja S.C. Mullick Road, Jadavpur, Kolkata 700032, India; 2 Developmental Therapeutics Branch and Laboratory of Molecular Pharmacology, Center for Cancer Research, National Cancer Institute, National Institutes of Health, Bethesda, MD 20892-4255, USA

## Abstract

Human tyrosyl-DNA phosphodiesterases (TDP) hydrolyze the phosphodiester bond between DNA and the catalytic tyrosine of Top1 to excise topoisomerase I cleavage complexes (Top1cc) that are trapped by camptothecin (CPT) and by genotoxic DNA alterations. Here we show that the protein arginine methyltransferase PRMT5 enhances the repair of Top1cc by direct binding to TDP1 and arginine dimethylation of TDP1 at residues R361 and R586. Top1-induced replication-mediated DNA damage induces TDP1 arginine methylation, enhancing its 3′- phosphodiesterase activity. TDP1 arginine methylation also increases XRCC1 association with TDP1 in response to CPT, and the recruitment of XRCC1 to Top1cc DNA damage foci. PRMT5 knockdown cells exhibit defective TDP1 activity with marked elevation in replication-coupled CPT-induced DNA damage and lethality. Finally, methylation of R361 and R586 stimulate TDP1 repair function and promote cell survival in response to CPT. Together, our findings provide evidence for the importance of PRMT5 for the post-translational regulation of TDP1 and repair of Top1cc.

## INTRODUCTION

DNA topoisomerase 1 (Top1) is essential for the release of DNA supercoiling generatedf during replication, transcription and chromatin remodeling (1,2). Supercoiling relaxation requires the production of reversible Top1-linked DNA single-strand breaks (SSBs) (Top1 cleavage complexes; Top1cc), which are normally transient but are selectively trapped by the anticancer drug camptothecin (CPT) and its clinical derivatives topotecan and irinotecan (2–4). Top1cc also accumulate under physiological conditions when Top1 acts on frequently occurring DNA alterations (mismatches, abasic sites, oxidized and adducted bases) (2,3,5). Trapping of Top1cc damages the genome by generating DNA double-strand breaks (DSBs) upon replication and transcription collisions (2), ensuing cell cycle arrest and cell death. Thus, repairing irreversible Top1cc is critical for DNA metabolism, genome maintenance and relevant to resistance of tumors to Top1 inhibitors (2,4–6).

Tyrosyl-DNA phosphodiesterase 1 (TDP1), the key enzyme for the repair of Top1cc, catalyzes the hydrolysis of the phosphodiester bond between the catalytic tyrosyl of Top1 and the 3′-end of DNA broken by Top1 (5). Genetic inactivation of TDP1 causes hypersensitivity to CPT (5,7–10). Homozygous mutation of TDP1 is also responsible for the neurodegenerative syndrome, spinocerebellar ataxia with axonal neuropathy SCAN1, which results from elevated levels of Top1cc in post-mitotic neurons (11–15). The importance of TDP1 outside Top1cc repair stems from the cleansing activity of TDP1 toward blocking DNA lesions at the 3′-end of DNA breaks, including phosphoglycolate, abasic sites, and alkylated bases at the 3′-end of DNA breaks (5,9,15–17) resulting from oxidative DNA damage produced by radiomimetic drugs such as bleomycin, alkylating agents and nucleoside analogs (5,7,9,17,18). TDP1 possesses nucleosidase activity for 3′-deoxyriboses, 3′-ribonucleotides and 3′-chain terminating anticancer and antiviral nucleosides (cytarabine, acyclovir, AZT and abacavir) and even 5′-phosphodiesterase activity for topoisomerase II cleavage complexes (5,17,19–21) and acts both in the cell nucleus and mitochondria (9,18).

The regulation of cellular TDP1 occurs mainly at the post-translational level (5,10). ATM-and/or DNA-dependent protein kinase (DNA-PK)-mediated S81 phosphorylation stabilizes TDP1 (10,22) and fosters the recruitment and activity of TDP1 for repairing Top1cc and ionizing radiation (IR)-induced DSBs (6,10,22–24). Poly(ADP-ribosyl)ation of TDP1 by poly(ADP-ribose) polymerase-1 (PARP1) also enhances the stability of TDP1 and its interaction with X-ray cross-complementing group 1 (XRCC1) and the recruitment of TDP1 to Top1cc damage sites (19). Additionally, SUMOylation of TDP1 at lysine 111 has been proposed to recruit TDP1 at transcription-associated Top1cc damage sites (25). The diversity of TDP1 post-translational modifications (PTMs) suggests that TDP1 is regulated through multiple cooperative events. However until now, none of the PTMs had any impact on the catalytic activity of TDP1 (10,19,22,25).

Arginine methylation is increasingly recognized as a pivotal post-translational modification orchestrating a variety of cellular processes including epigenetic regulation, DNA repair and genome maintenance (26–29). It is carried out by protein arginine methyltransferases (PRMTs) that catalyze the methylation of the guanidium group of arginine residues using S-adenosyl methionine (SAM) as a methyl group donor. PRMTs are classified as type 1 (PRMT1, PRMT2, PRMT3, PRMT4, PRMT6, PRMT8), type 2 (PRMT5 and PRMT9) and type 3 (PRMT7) enzymes on the basis of their ability to catalyze the formation of asymmetric (ADMA), symmetric dimethylated arginine (SDMA) and monomethylated arginine (MMA), respectively (30). Until this report, arginine methylation had not been implicated in the cellular responses to Top1cc.

Human PRMT5 is commonly activated in cancers. It stimulates cellular proliferation by adding SDMA marks on a range of acceptor proteins including the core histones H3 and H4, leading to transcription repression of tumor suppressor genes (RB1 and CUL4A), and by adding SDMA activating marks on non-histone proteins including p53, E2F1 and two DNA repair proteins FEN1 and RAD9 associated with DNA replication (26–28,31–34). Our study provides the first evidence that PRMT5 is a molecular determinant for Top1cc repair. We show that TDP1 is dimethylated at R361 and R586 by PRMT5, and that arginine methylation of TDP1 is a critical modulator of the catalytic activity of TDP1, and of its association with XRCC1 for the repair of Top1cc-mediated DNA damage.

## MATERIALS AND METHODS

### Drug and antibodies

Camptothecin (CPT), aphidicolin (APH), propidium iodide (PI) and, 5,6-dichlorobenzimidazole 1-β-d-ribofuranoside (DRB) were purchased from Sigma (St Louis, MO, USA). Rabbit polyclonal anti-symmetric dimethyl arginine (SDMA) (SYM10 and SYM11), anti-PRMT5 (07-405), anti-PRMT9 (MABE1112) and mouse monoclonal anti-γH2AX (05-636) antibodies were purchased from Millipore, USA. Rabbit polyclonal TDP1 (Ab4166) and GAPDH (Ab9485), mouse monoclonal XRCC1 (Ab1838) and Histone H3 (Ab24834) antibodies were purchased from Abcam (Cambridge, MA, USA). Mouse monoclonal anti-flag (M2) (F3165), rabbit polyclonal anti-FLAG (F7425) antibodies were purchased from Sigma (St Louis, MO, USA). The anti-PAR rabbit polyclonal antibody was from Trevigen (Gaithersburg, MD, USA). Anti-actin (ACTN05) antibody was from Neo Markers (Fremont, CA, USA). Rabbit polyclonal anti-GFP (A-11122) antibody was from Invitrogen. Rabbit polyclonal PARP1 antibody and secondary antibodies: Horseradish peroxidase (HRP)-conjugated anti-rabbit IgG or anti-mouse IgG were obtained from Santa Cruz Biotechnology (Santa Cruz, CA, USA).

### Expression constructs and site-directed mutagenesis

Human flag-tagged full-length TDP1 (FLAG-TDP1^WT^), His-tagged and green fluorescent protein (GFP)-tagged TDP1 constructs were described previously (10,19). The FLAG-PRMT5 fusion construct was a kind gift from Dr Shilai Bao (Institute of Genetics and Developmental Biology, CAS, China). The flag-tagged N-terminal (1–293 aa) and C-terminal (294–637 aa) truncated PRMT5 and GFP-tagged N-terminal (1–185 aa) truncated TDP1 constructs were generated by polymerase chain reaction amplification using full-length PRMT5 or full-length TDP1 (FLAG-TDP1^WT^) as template and were cloned in the mammalian expression vectors pCMV-Tag2 (Stratagene, La Jolla, CA, USA) or pEGFP-N2 vector (CLONTECH) respectively. The following point mutations: TDP1^R361K^, TDP1^R586K^, TDP1^R361K, R586K^ in FLAG and GFP tagged TDP1 constructs as well as His-TDP1^R361K,R586K^ were created using the ‘QuickChange’ protocol (Stratagene, La Jolla, CA, USA). All PCR-generated constructs were confirmed by DNA sequencing.

### Cell culture, treatment and transfections

Cell cultures were maintained at 37°C under 5% CO_2_ in Dulbecco's modified Eagle's medium containing 10% fetal calf serum (Life Technologies, Rockville, MD, USA). The colon carcinoma cell line (HCT116), human kidney origin (HEK293) and human breast cancer (MCF7) was obtained from the Developmental Therapeutics Program (NCI, NIH/ USA). TDP1^+/+^ and TDP1^−/−^ primary MEF cells were a kind gift from Dr Cornelius F Boerkoel (University of British Columbia, Vancouver, British Columbia, Canada). Cells were treated with the indicated concentrations of CPT. Plasmid DNAs were transfected with Lipofectamine 2000 (Invitrogen) according to the manufacturer's protocol. TDP1^−/−^ MEF cells were transfected with the FLAG-TDP1 constructs using X-tremeGENE HP DNA transfection reagent (Roche) according to the manufacturer's protocol.

### siRNA transfection

Transfections were performed as described previously (10). In brief, cells (1.5 × 10^5^) were transfected with control siRNA or 25 nM PRMT5 siRNA (GE Dharmacon, SiRNA-SMARTpool) using oligofectamine (Invitrogen) according to the manufacturer's protocol. Time course experiments revealed a maximum suppression of PRMT5 protein at day 3 after transfection, as analyzed by western blotting.

### Cell extracts, immunoblotting, and immunoprecipitation

Preparation of whole cell extracts, immunoprecipitation, and immunoblotting were carried out as described previously (10,18,19). Briefly, cells were lysed in a lysis buffer (10 mM Tris–HCl (pH 8), 150 mM NaCl, 0.1% SDS, 1% NP40, 0.5% Na-deoxycholate supplemented with complete protease inhibitors) (Roche Diagnostics, Indianapolis, IN) and phosphatase inhibitors (Phosphatase Inhibitor Cocktail 1 from Sigma). After thorough mixing and incubation at 4°C for 2 h, lysates were centrifuged at 12 000 g at 4°C for 20 min. Supernatants were collected, aliquoted, and stored at –80°C.

For immunoprecipitation, cells were lysed in a lysis buffer (50 mM Tris–HCl (pH 7.4), 300 mM NaCl, 0.4% NP40, 10 mM MgCl_2_, 0.5 mM dithiothreitol supplemented with protease and phosphatase inhibitors). Supernatants of cell lysates were obtained by centrifugation at 15 000 g at 4°C for 20 min and pre-cleared with 50 μl of protein A/G-PLUS agarose beads (Santa Cruz, CA, USA). About 5 mg of pre-cleared lysate was incubated overnight at 4°C with indicated antibodies (2–5 μg/ml) and 50 μl of protein A/G-PLUS agarose beads. Isolated immunocomplexes were recovered by centrifugation, washed thrice with lysis buffer, and were subjected to electrophoresis on 10% Tris–glycine gels and immunoblot analysis. Immunoblottings were carried out following standard procedures, and immunoreactivity was detected using ECL chemiluminescence reaction (Amersham) under ChemiDoc™ MP System (Bio-Rad, USA). Densitometric analyses of immunoblots were performed using Image J software.

### Immunocytochemistry and confocal microscopy

Immunofluorescence staining and confocal microscopy were performed as described previously (10,18,19). Briefly, cells were grown and drug treated on chamber slides (Thermo Scientific™ Nunc™ Lab-Tek™ II Chamber slides) followed by fixation with 4% paraformaldehyde for 10 min at room temperature. Primary antibodies against PRMT5, γH2AX and XRCC1 were detected using anti-rabbit or anti-mouse IgG secondary antibodies labeled with Alexa 488/568 (Invitrogen). Cells were mounted in anti-fade solution with 4′,6-diamidino-2-phenylindole (DAPI) (Vector Laboratories, Burlingame, CA, USA) and examined under Leica TCS SP8 confocal laser-scanning microscope (Germany) with a 63×/1.4 NA oil objective. Images were collected and processed using the Leica software and sized in Adobe Photoshop 7.0. The γH2AX intensity per nucleus was determined with Adobe Photoshop 7.0 by measuring the fluorescence intensities normalized to the number of cell count (10,18,19).

### *In vitro* methylation assays

The *in vitro* methyaltion assays were carried out as described previously (31,33,34). Briefly, PRMT5 was immunoprecipitated and incubated with recombinant His-TDP1^WT^ or His-TDP1^KK^ proteins (1.5 μg). Methylation reactions were carried out using methylation buffer (50 mM Tris–HCl, (pH 8.5), 5 mM MgCl_2_, 4 mM DTT) containing 100 μM unlabeled *S*-(5′-adenosyl)-l-methionine chloride dihydrochloride (SAM) (A7007) (Sigma) for 2 hours at 30°C. Reactions were stopped by adding 2× SDS loading buffer (Invitrogen) and boiling the samples for 5 minutes. Methylation reaction products were separated by SDS–PAGE, transferred on to PVDF membrane and analyzed by Western blotting using anti-SDMA and anti-TDP1 antibodies.

### Oligonucleotides and preparation of DNA substrates

The N14Y oligonucleotide (5′-GATCTAAAAGACTT**Y**-3′), which contains a 3′-phosphotyrosine (**Y**) was synthesized by Midland Certified Reagents Company (Midland, TX, USA). The N14Y oligonucleotide was 5′-end labeled using T4 polynucleotide kinase and [γ-^32^P] ATP. Unincorporated radioactive nucleotides were removed using a mini Quick Spin Oligo column (Roche Diagnostics) after inactivation of the kinase by heating for 5 min at 95°C.

### TDP1 activity assays

TDP1 activity assays were performed as described previously (9,18,35). Briefly, cellular lysates obtained from TDP1^−/−^ MEF cells transfected with FLAG–TDP1^WT^, FLAG–TDP1^KK^ or vector control, and, PRMT5 or control siRNA transfected HCT116 cells were subjected for gel based TDP1 assay. Additionally, *in vitro* methyaltion of His-tagged TDP1 was carried out as described above and were subjected for gel based TDP1 assays. HCT116 cells transfected with FLAG or GFP–TDP1^WT^ or FLAG or GFP–TDP1^KK^ were immunoprecipitated using anti-FLAG or anti-GFP antibody and the purified immune complexes were used as source of TDP1 activity. One nanomolar of the 5′-end radiolabeled N14Y substrate was incubated either with the cell lysates or purified immune complexes for 30 min at 25°C in a reaction buffer containing 1× PBS, 80 mM KCl, and 0.01% Tween-20. Reactions were terminated by the addition of two volumes of gel loading buffer (96% (v/v) formamide, 10 mM EDTA, 1% (w/v) xylene cyanol and 1% (w/v) bromophenol blue). The samples were subsequently heated for 5 min at 95°C and subjected to 20% sequencing gel electrophoresis. Gels were then dried and exposed on PhosphorImager screens. Imaging and quantification were done using Typhoon FLA 7000 and ImageQuant software (GE Healthcare, UK). TDP1 activity was determined by measuring the percentage of 14Y converted to 14P by densitometry analysis of the gel image.

### Alkaline COMET assays

To compare the levels of DNA damage in PRMT5 depleted cells and TDP1^−/−^ MEFs cells transfected with FLAG-TDP1^WT^, FLAG-TDP1^KK^ and vector control, were subjected to alkaline comet assays according to the manufacturer's instructions (Trevigen, Gaithesburg, MD) as described previously (10,13,36). Briefly, after treatment with 5 μM CPT, cells were collected and mixed with low melting agarose. Slides were immersed in lysis solution at 4°C for 1 h. After a rinse with deionized water, slides were immersed in a 4°C alkaline solution (50 mM NaOH, 1 mM EDTA, and 1% dimethyl sulfoxide) for 1 h. Electrophoresis was carried out at a constant voltage of 25 V for 30 min at 4°C. After electrophoresis, slides were neutralized in 0.4 M Tris–HCl (pH 7.5), dehydrated in ice-cold 70% ethanol for 5 min, and air-dried. DNA was stained with ethidium bromide (EtBr) purchased from Sigma (USA). The relative length and intensity of EtBr-stained DNA, tails to heads, is proportional to the amount of DNA damage present in the individual nucleus. Comet length was measured using the TriTek Comet Score software (TriTek Corp, Sumerduck, VA) and was scored for at least 50 cells. Distributions of comet lengths were compared using the Student *t*-test.

### Cell survival assays

Cells (6 × 10^3^) were transfected with control or PRMT5 siRNA (25 nM) as described above and seeded in 96-well plates (BD Biosciences, USA). After 24 h, cells were treated with CPT at the indicated concentrations and kept further for 48 h. Cell survival was then assessed by 3-(4,5-dimethylthiazol-2-yl)-2,5-diphenyltetrazolium bromide (MTT) purchased from Sigma, USA as described previously (37). Plates were analyzed on Molecular Devices SpectraMax M2 Microplate Reader at 570 nm. The percent inhibition of viability for each concentration of CPT was calculated with respect to the control. Data represent mean values ± S.D. for three independent experiments.

For the clonogenic assays (10), TDP1^−/−^MEF cells (2 × 10^6^) were separately transfected with 5 μg of plasmid DNA (FLAG–TDP1^WT^, FLAG–TDP1^KK^, or vector control) using X-tremeGENE HP DNA transfection reagent (Roche) according to the manufacturer's protocol, protein expressions were determined by Western blot analysis. After 5 h treatment with the indicated concentrations of CPT, cells were trypsinized, washed in PBS, and seeded in triplicate at a density of 500 cells per well in six-well plates. Colonies were allowed to grow for 10–12 days and visualized after washing with PBS, fixation in methanol for 30 min, washing again with PBS, and staining with 0.05% methylene blue for 30 min. Percent survival was normalized to the observed number of colonies generated from untreated cells (14). Data represent mean values ± S.E.M. for three independent experiments.

### Mass spectrometry analysis of TDP1

Ectopic FLAG-TDP1 complexes were immunoprecipitated with anti-FLAG antibody as described above. To induce DNA damage cells expressing FLAG-TDP1 were treated with CPT (5 μM/3 h) prior to anti-FLAG immunoprecipitation and were subjected to tryptic digestion at 37°C, overnight, followed by lyophilization, reconstitution, and fractionation applying strong cation exchange (SCX) liquid chromatography (LC) and mass spectrometry analysis as previously described (38).

### Cell cycle analysis

Cell cycle analysis was performed as described previously (31). Briefly cells (1 × 10^6^) were transfected with control or PRMT5 siRNA (25 nM) as described above and seeded in six-well plates. After 48 h, cells were treated with 5 μg / ml aphidicolin (Sigma) and kept further for 24 h. Cells were then harvested, rinsed in PBS, permeabilised in ice cold 70% ethanol, and kept at 4°C overnight. Prior to fluorescence-activated cell-sorting (FACS) analysis, cells were washed with PBS and stained with 500 μl of propidium iodide (PI) solution containing 10 μg/ml PI and 100 μg/ml RNase A (Sigma). Following incubation in the dark at room temperature for 45 minutes, the samples were analyzed on a FACS Calibur (Becton Dickinson), and the percentages of G0/G1, S and G2/M populations were determined using BD FACS Diva 8.0.1 software.

### Cell fractionation and isolation of chromatin bound protein

For cell fractionation and isolation of chromatin bound proteins (39), cells were washed with 1× PBS followed by washing with hypotonic buffer containing 20 mM HEPES, pH 7.5, 20 mM NaCl, 5 mM MgCl_2_ and suspended in hypotonic buffer (10 ml). Post 10 min incubation on ice, cells were lysed to free nuclei by 45 strokes of a dounce homogenizer and were centrifuged at 1500 g at 4°C for 5 min to isolate the supernatant from the nuclear pellet. Nuclei were further suspended in extraction buffer containing 50 mM HEPES, pH 7.5, 100 mM KCl, 0.25% Triton X-100, 2.5 mM MgCl_2_, 1mM dithiothreitol, aprotinine (1 μM), leupeptine (50 μM), 4-(2-aminoethyl)-bezenesulfonylfluoride/HCl (1 mM) and NaF (10 mM) followed by centrifugation at 600 g at 4°C for 3 min. Nuclei were further suspended thrice in extraction buffer for complete lysis of the nuclear envelope and full extraction. Supernatants were pooled to yield nucleosolic proteins and the residual pellet contained all DNA and structure bound proteins (chromatin fraction).

## RESULTS

### PRMT5 physically interacts with TDP1

**Figure 1.**
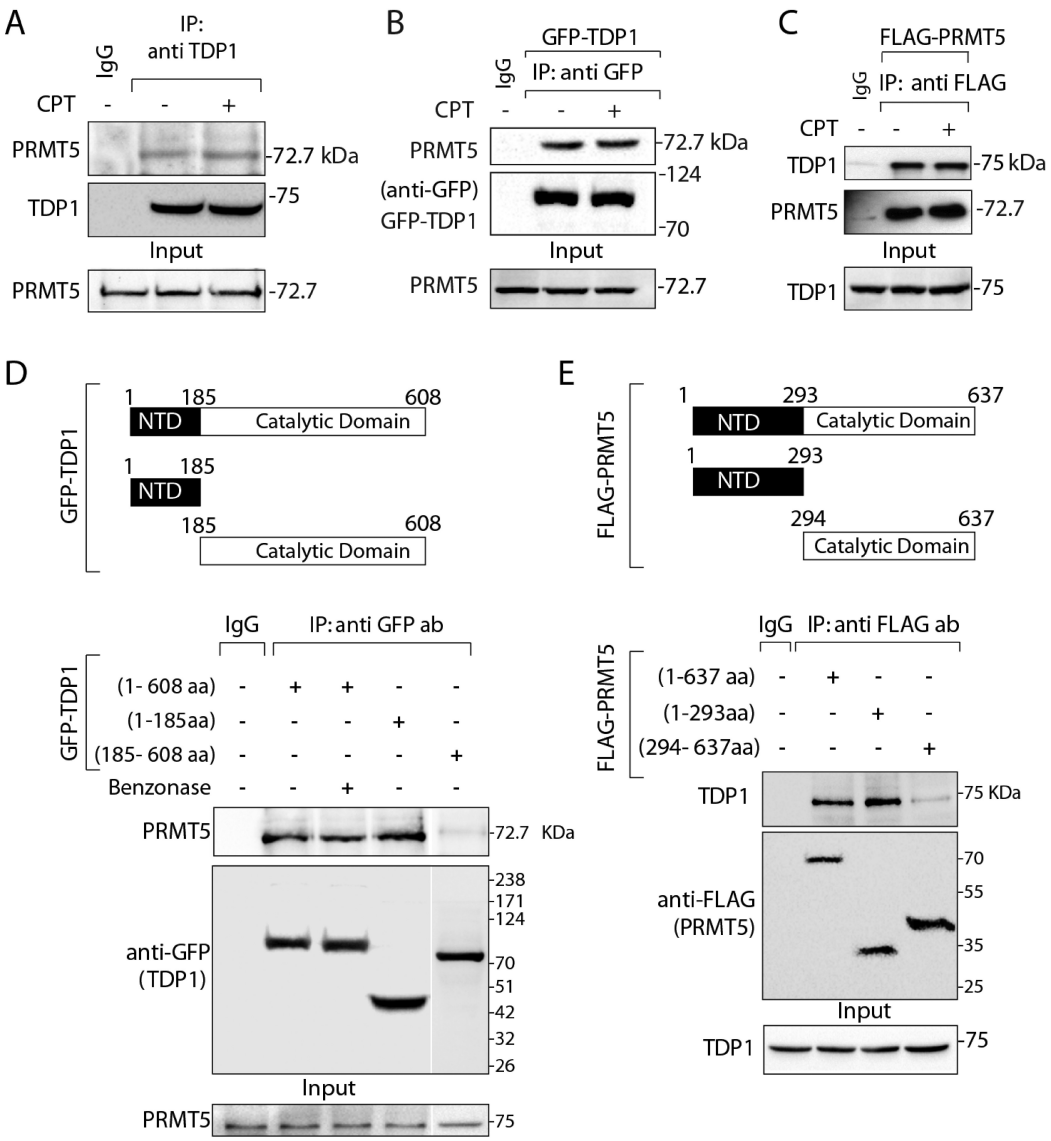
PRMT5 physically interacts with TDP1. (**A**) Endogenous TDP1 from HCT116 cells treated with or without CPT (5 μM, 3 h) was immunoprecipitated using anti-TDP1 antibody and the immune complexes were blotted with anti-PRMT5 antibody. The same blot was stripped and reprobed with anti-TDP1 antibody. Aliquots (10%) of the input show the level of PRMT5 prior to immunoprecipitation. (**B**) HCT116 cells ectopically expressing GFP-TDP1 treated with or without CPT (5 μM, 3 h), and were immunoprecipitated using anti-GFP antibody. Immune complexes were blotted with anti-PRMT5 antibody. The same blot was stripped and reprobed with anti-GFP antibody to show the expression of the GFP-TDP1. Aliquots (10%) of the input show the level of PRMT5 prior to immunoprecipitation. (**C**) HCT116 cells ectopically expressing FLAG-PRMT5 were immunoprecipitated using anti-flag antibody and the immune complexes were blotted with anti-TDP1 antibody. The same blot was stripped and reprobed with anti-PRMT5 antibody. Aliquots (10%) of the input show the level of TDP1 prior to immunoprecipitation. (**D**) Schematic representation of GFP-tagged constructs showing full length (1-608 aa), truncated N-terminal domain (1–185 aa; N-terminal domain [NTD]), and truncated C-terminal domain (185–608 aa; catalytic domain) of human TDP1 are indicated. Ectopic GFP-TDP1 variants were expressed in HCT116 cells and immunoprecipitated using anti-GFP antibody and the immune complexes were probed with anti-PRMT5 antibody. To examine direct protein-protein interaction cell lysates were pretreated with benzonase prior to co-IP as indicated. Blots were subsequently stripped and probed with anti-GFP antibody to show the expression of the GFP-TDP1 variants. Aliquots (10%) of the input show the level of PRMT5 prior to immunoprecipitation. Migration of protein molecular weight markers (kDa) is indicated at right. (**E**) Schematic representation of flag-tagged constructs showing full length (1–637 aa), truncated N-terminal domain (1–293 aa) and truncated C-terminal domain (294–637 aa) of human PRMT5. Flag-tagged PRMT5 constructs were ectopically expressed in HCT116 cells and were co-immunoprecipitated with anti-flag antibody. The immune complexes were probed with anti-TDP1 antibodies. Blots were subsequently stripped and probed with anti-flag antibody to show the expression of FL and truncated constructs of Flag-PRMT5. Aliquots (10%) of the input show the level of TDP1 prior to immunoprecipitation. Migration of protein molecular weight markers (kDa) is indicated at right.

The emerging role of PRMT5 in the DNA damage response pathways (27,28,31–34) prompted us to test its role in Top1cc repair. Because TDP1 is the key repair protein for Top1cc, we directly examined TDP1-PRMT5 interaction. We pulled down endogenous TDP1 from HCT116 cells and tested TDP1-PRMT5 association. Co-immunoprecipitation (co-IP) of endogenous TDP1 pulled down endogenous PRMT5 (Figure 1A) both in the presence and absence of CPT, indicating TDP1-PRMT5 binding independent of DNA damage. Figure 1B shows endogenous PRMT5 in the GFP-TDP1 co-immunoprecipitation in cells ectopically expressing GFP-TDP1 (Figure 1B) both in the presence and absence of CPT. We further established the presence of TDP1 in the PRMT5-complex using reverse co-IP in cells ectopically expressing FLAG-PRMT5 (Figure 1C). We also confirmed that the TDP1-PRMT5 association is independent of the TDP1 fusion tag by pulling down ectopic FLAG-tagged TDP1 with an anti-FLAG antibody both in the presence and absence of CPT (Supplementary Figure S1A). Under similar condition we did not detect PRMT9, another type 2 arginine methyltransferases in the GFP-TDP1 co-immunoprecipitation (Supplementary Figure S1B), confirming the specific association between TDP1 and PRMT5.

To identify the interacting domains between TDP1 and PRMT5, we used the GFP-tagged fragments of TDP1 (Figure 1D) and flag-tagged PRMT5 (Figure 1E) corresponding to their different domains. The GFP-tagged N-terminal domain of TDP1 (1–185 amino acids) was sufficient to pull down endogenous PRMT5. We also detected a weak binding of PRMT5 with the catalytic domain of TDP1 (185–608 amino acids) indicating that the catalytic domain of TDP1 is not necessary for the interaction of TDP1 with PRMT5. We also observed a weak binding of PRMT5 with the C-terminal domain of TDP1 (185-608 amino acids). To test whether PRMT5 directly interacts with TDP1, we performed co-IP with GFP-TDP1 in the presence of the benzonase nuclease. We found that the TDP1-PRMT5 association was resistant to benzonase, indicating a direct protein-protein interaction, not mediated through DNA (Figure 1D).

Next, to determine the domain of PRMT5 interacting with TDP1, we used truncated flag-tagged N- and C-terminal domains of PRMT5 as shown in Figure 1E (40). Flag-pull down experiments with N-terminal domain of PRMT5 (1-293 amino acids) detected endogenous TDP1 (Figure 1E). We also observed a weak binding of TDP1 with the C-terminal domain of PRMT5 (294–637 amino acids), which was predominantly distributed in the cytoplasmic soluble fraction of HCT116 cells similar to the N-terminal domain of PRMT5 (1–293 amino acids) and full-length PRMT5 (Supplementary Figure S1D). The C-terminal domain (294–637 amino acids) of PRMT5 contains the catalytic domain (40). Therefore, it is conceivable that PRMT5 interacts with TDP1 through its N-terminal domain without interfering its C-terminal catalytic domain.

### PRMT5 catalyzes TDP1 methylation at R361 and R586

**Figure 2.**
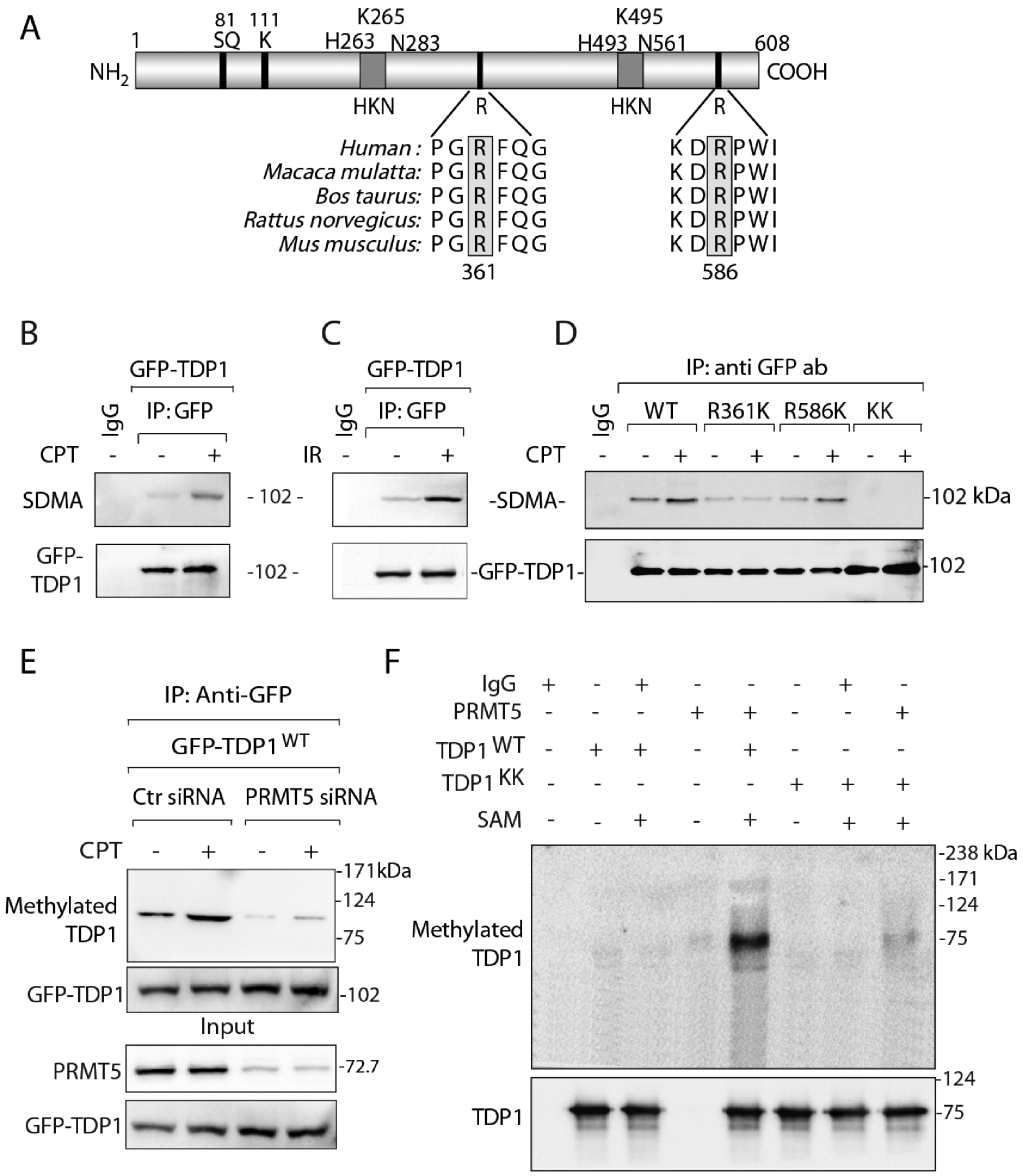
TDP1 is methylated at R361 and R586 by PRMT5. (**A**) Schematic representation of human TDP1 showing the arginine dimethylation sites (R361 and R586), the S81-phosphorylation site, the K111-SUMOylation site and the catalytic residues (HKN motifs). Alignment of TDP1 sequences spanning R361 and R586 (highlighted in grey boxes) from human (*Homo sapiens*), monkey (*Macaca mulatta*), cattle (*Bos taurus*), rat (*Rattus norvegicus*), mouse (*Mus musculus*) demonstrates their phylogenetic conservation. (**B**) HCT116 cells ectopically expressing GFP-TDP1 were treated with or without CPT (5 μM, 3 h). GFP-TDP1 was immunoprecipitated using anti-GFP antibody and the immune complexes were blotted with SDMA-specific antibody. The same blot was stripped and reprobed with anti-GFP antibody to show equal loading. (**C**) HCT116 cells ectopically expressing GFP-TDP1 were treated with IR (10 Gy). Cells were analyzed 3 h after irradiation. GFP-TDP1 was immunoprecipitated using anti-GFP antibody and the immune complexes were blotted with SDMA-specific antibody. The same blot was stripped and reprobed with anti-GFP antibody to show equal loading. (**D**) Detection of arginine methylation of TDP1 at R361 and R586. The GFP-tagged TDP1 constructs: wild-type (GFP-TDP1^WT^), single-mutants for arginine methylation sites: GFP-TDP1^R361K^ and GFP-TDP1^R586K^, and the double-mutant R361K + R586K [KK] were ectopically expressed in HCT116 cells, treated as indicated with CPT (5 μM, 3 h). GFP-TDP1 variants were immunoprecipitated using anti-GFP antibody and the immune complexes were blotted with anti-SDMA-specific antibody. The same blot was stripped and reprobed with anti-GFP antibody to show equal loading. Control immunoprecipitation with anti-IgG demonstrates the specificity of the reactions. Migration of protein molecular weight markers (kDa) is indicated at right. (**E**) PRMT5 depletion abrogates the symmetric dimethylation of arginine residues on TDP1. HCT116 cells were transfected with PRMT5 or control (Ctr) siRNA, then transfected 48 h later with a GFP-tagged human TDP1 construct (GFP-TDP1^WT^). Following CPT treatment (5 μM, 3 h), ectopic GFP-TDP1 was immunoprecipitated using anti-GFP antibody and the immune complexes were blotted with SDMA specific antibodies. The same blot was stripped and reprobed with anti-GFP antibody. Aliquots (10%) of the input show the level of PRMT5 knockdown, and GFP-TDP1 prior to immunoprecipitation. Electrophoretic migration of protein molecular weight markers (kDa) is indicated at right. (**F**) *In vitro* methylation assay with flag-tagged PRMT5 immunoprecipitated from HCT116 cells using anti-flag antibody with unlabeled S-adenosylmethionine (SAM). The substrates were recombinant His-tagged TDP1: wild-type (WT) and double-mutant for the R361 and R586 methylation sites (KK). The same blot was stripped and reprobed with anti-TDP1 antibody showing the amount of substrate in each reaction. Migration of protein molecular weight markers (kDa) is indicated at right.

To investigate the significance of TDP1-PRMT5 association we examined TDP1 methylation using mass spectrometry (MS). MS analysis of FLAG-TDP1 immunoprecipitation complex detected R361 and R586 as dimethylated arginine residues on TDP1 (Supplementary Figure S2). MS data also revealed that TDP1-R586 dimethylation was detected independently of DNA damage, while CPT triggered TDP1-R361 dimethylation, indicating that DNA damage enhances TDP1 arginine methylation. Both R361 and R586 of human TDP1 are phylogenetically conserved across vertebrate species (Figure 2A), and R361 is within a conserved motif, which is the preferred substrate for PRMT5 (26).

To confirm TDP1 arginine methylation by PRMT5 (Supplementary Figure S2), we performed co-immunoprecipitation of ectopic GFP-TDP1 in cells treated with or without CPT and probed with antibodies that recognize symmetrically dimethylated arginine residues (anti-SDMA) (31,41,42). Figure 2B shows that GFP-TDP1 reacts to the SDMA specific antibody. The methylation signal on TDP1 was consistently increased (∼40%) upon CPT treatment (Figure 2B; D and E). TDP1 arginine dimethylation was induced both by Top1cc (CPT) and ionizing radiation (Figure 2C). To further validate the methylation of TDP1 on its R361 and R586 residues, we ectopically expressed methylation mutant GFP-TDP1 variants (single mutants: R361K and R586K, and the double-mutant: R361K + R586K [KK]) (Figure 2D). The TDP1 single mutant GFP-TDP1^R361K^ showed strong reduction and the single mutant GFP-TDP1^R586K^ a weaker reduction, while the double-mutant (GFP-TDP1^KK^) abolished the methylation signal on TDP1, confirming selective TDP1 methylation on R361 and R586 residues (Figure 2D). Our data demonstrate that DNA damage (CPT or IR) induce TDP1 arginine methylation on residues 361 and 586 (Figure 2B, C and D).

To establish whether PRMT5 is responsible for TDP1 arginine methylation, we ectopically expressed GFP-tagged wild-type TDP1 in PRMT5-knockdown cells using small interfering RNA (siRNA). Figure 2E shows that PRMT5 depletion resulted in a marked decrease in arginine-methylated TDP1, showing that TDP1 not only physically interacts (see Figure 1) but is also arginine methylated *in vivo* by PRMT5.

To obtain further evidence for TDP1 methylation by PRMT5 at R361 and R586, we performed *in vitro* methylation assays with recombinant His-tagged TDP1 (WT [TDP1^WT^] and double-mutant R361K + R586K [TDP1^KK^]) as substrates for immunoprecipitated PRMT5 in the presence of S-adenosylmethionine (SAM). Methylation of TDP1^WT^ by PRMT5 was suppressed in the TDP1^KK^ double-mutant (Figure 2F), demonstrating that R361 and R586 are the major residues for PRMT5-mediated TDP1 arginine methylation. Under similar condition PRMT9 failed to methylate the TDP1 arginine residues (Supplementary Figure S1C), affirming that PRMT5 is the key arginine methyltransferase for TDP1. Because R361 and R586 (Figure 2A) as well as PRMT5 are conserved among vertebrates (26), we conclude that R361 and R586 of TDP1 are plausible cellular targets for PRMT5 across species.

### PRMT5 depletion enhances Top1-induced DNA damage

**Figure 3.**
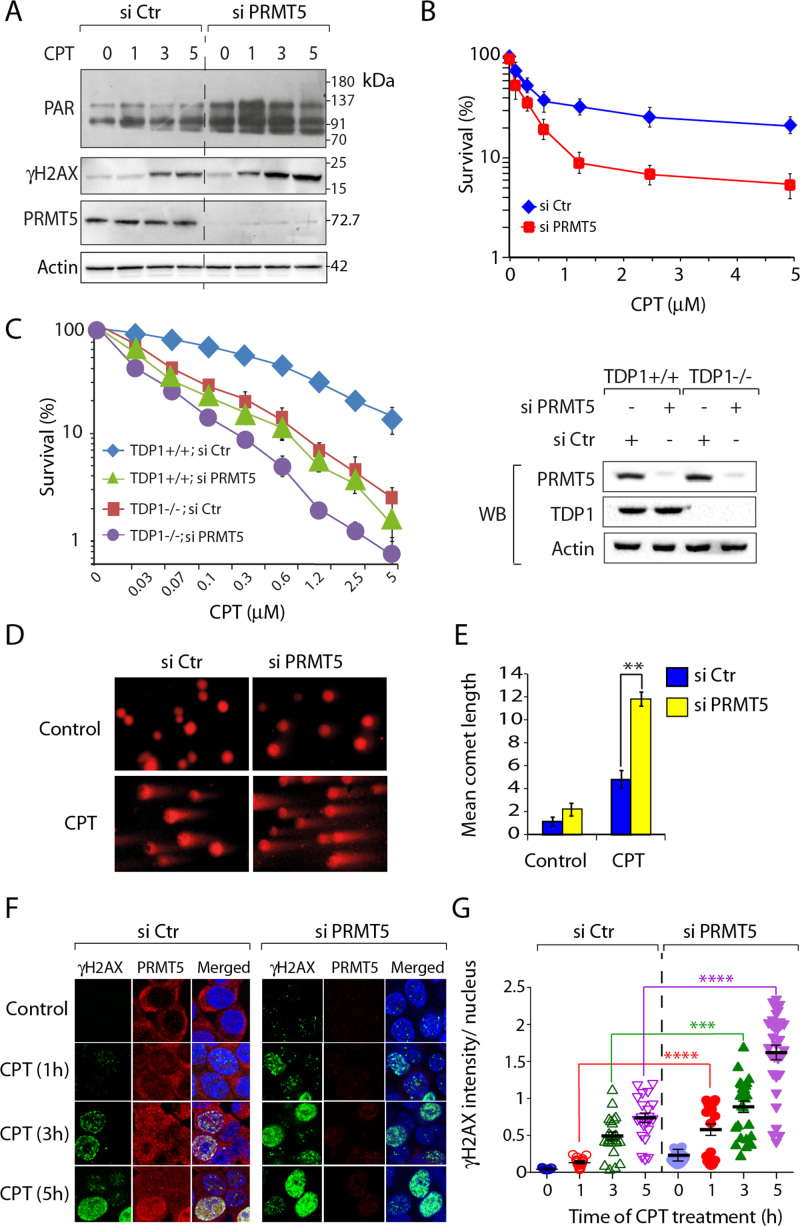
PRMT5 deficient cells are hypersensitive to camptothecin. (**A**) siRNA knockdown of PRMT5 enhances CPT-induced DNA damage response. Following transfection with PRMT5 or control siRNA for 72 h, HCT116 cells were treated with CPT (5 μM) for the indicated times (h), and protein levels (PAR, γH2AX and PRMT5) were analyzed by western blotting (A representative experiment is shown). Actin served as loading control. Migration of protein molecular weight markers (kDa) is indicated at right. (**B**) Cell survival curves of HCT116 cells transfected with PRMT5 or control siRNA. CPT-induced cytotoxicity (%) was calculated with respect to the untreated control. Each point corresponds to the mean ± S.D. of at least three experiments. Error bars represent SD (*n* = 3). (**C**) Cell survival curves of TDP1^+/+^ and TDP1^−/−^ MEF cells transfected with PRMT5 or control siRNA. CPT-induced cytotoxicity (%) was calculated with respect to the untreated control. Each point corresponds to the mean ± S.D. of at least three experiments. Error bars represent SD (*n* = 3). Western blots showing siRNA-mediated depletion of PRMT5 in TDP1^+/+^ and TDP1^−/−^ MEF cells. (**D**) PRMT5 depletion produces an accumulation of CPT-induced DNA strand breaks. Representative images of alkaline comet assays in control and PRMT5-depleted HCT116 cells treated with CPT (5 μM, 1 h). (**E**) Quantification of CPT-induced DNA strand breaks calculated for 20–25 cells (average ± S.E.M). Asterisks denote significant difference (**P<0.001; *t* test) between control and PRMT5-depleted cells. (**F**) PRMT5 depletion enhances CPT-induced γH2AX. Confocal immunofluorescence microscopic analysis of CPT (5 μM)-induced γH2AX in control and PRMT5-depleted HCT116 cells after the indicated times. PRMT5 and γH2AX are shown in red and green respectively. Nuclei were stained with DAPI (blue). (**G**) Quantification of CPT-induced γH2AX intensity per nucleus obtained from confocal immunofluorescence microscopy was calculated for 20–25 cells (calculated value ± S.E.M.) and plotted as a function of time (h). Asterisks denote significant difference (****P < 0.0001; *t* test) in CPT-induced γH2AX intensity between control and PRMT5 depleted cells. Note: CPT-induced accumulation of PRMT5 in the nucleus.

To test the mechanistic link between PRMT5 and Top1-induced DNA damage, we measured ADP-ribose polymers (PAR) and the DSB marker γH2AX (10,19) in PRMT5 knockdown and proficient cells. Both PAR and γH2AX were consistently increased (∼3-fold) in PRMT5-deficient cells treated with CPT (Figure 3A), suggesting a role of PRMT5 in limiting Top1cc-induced DNA damage.

To further establish the role of PRMT5 in Top1cc repair, we performed survival assays. Figure 3B shows that inactivation of PRMT5 increased the cytotoxicity of CPT in human colon carcinoma HCT116 cells. Similarly, knocking down PRMT5 in HEK293 and MCF7 human cells (Supplementary Figure S3A and B) or in mouse embryonic fibroblast led to a marked increase in CPT-induced cytotoxicity (Figure 3C), implying that the protective role of PRMT5 is independent of tissue types. Genetic inactivation of TDP1 or PRMT5 in MEFs cells induces increased cytotoxicity to CPT (Figure 3C), and we observed an additional sensitivity to CPT (Figure 3C) upon double inactivation of TDP1 plus PRMT5 in mouse cells (TDP1-/- / siPRMT5), suggesting that PRMT5 exhibits additional mechanisms for the repair of Top1cc independently of TDP1.

Next, we used alkaline comet assays (Figure 3D and E) to compare the CPT-associated DNA strand breaks (10,13,25) in PRMT5-deficient and -proficient cells. We measured the level of DNA strand breaks following 1 h incubations with CPT, and Figure 3E shows that CPT-treated PRMT5-deficient cells accumulate ∼3-fold more DNA breaks as compared to the control cells.

We further determined DNA damage in PRMT5-deficient cells as CPT-induced γH2AX foci at the single cell level with confocal immunofluorescence microscopy (10). Figure 3F shows representative images demonstrating enhanced CPT-induced γH2AX foci in PRMT5-depleted cells. Quantitation showed a ∼3-fold increase in γH2AX at all time points examined (Figure 3G), which demonstrates increased CPT-induced DNA damage in PRMT5-deficient cells (Figure 3B). Interestingly, under similar conditions, in PRMT5-proficient cells, CPT induced PRMT5 signals in the nucleus at DNA damage sites marked by γH2AX foci (Figure 3F; CPT 5 h). Consistently, CPT lead to an increased chromatin binding of PRMT5 (Supplementary Figure S1E). Taken together our data provide evidence for the engagement and role of PRMT5 in Top1cc repair.

### Coordinated role of PRMT5 and TDP1 for the repair Top1cc-induced replication-mediated DNA damage

**Figure 4.**
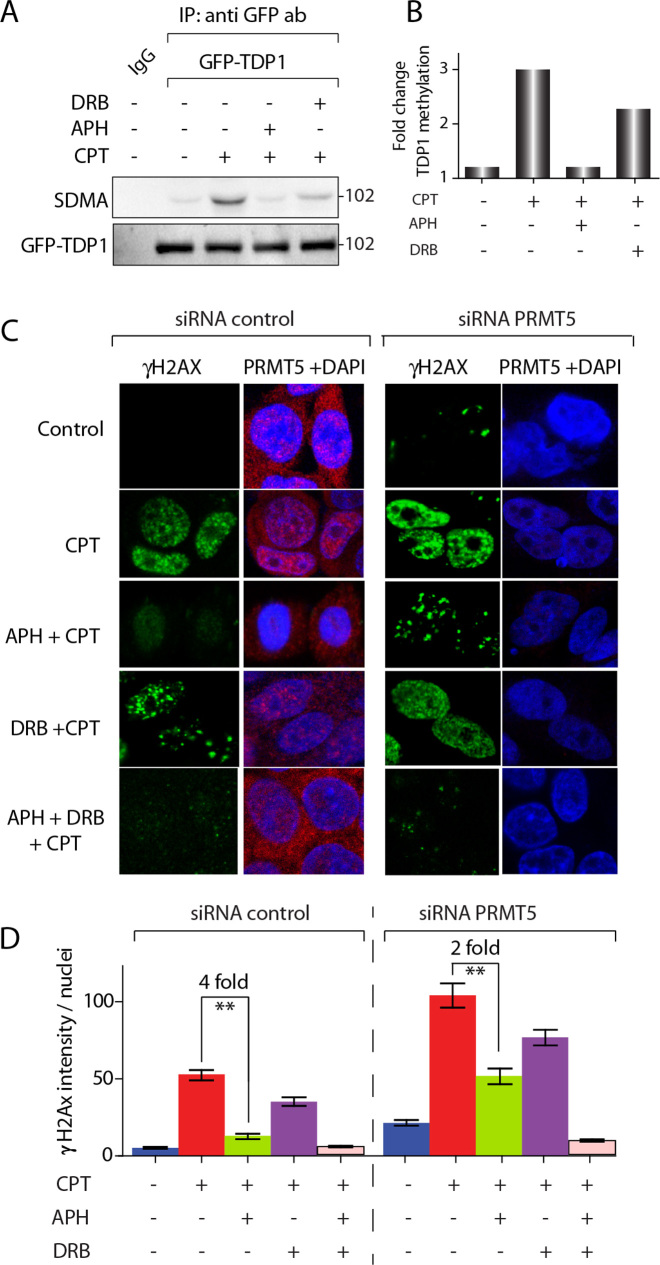
Replication-coupled DNA damage induces TDP1 arginine methylation and PRMT5-dependent Top1cc repair. (**A**) TDP1 methylation induction by replication DNA damage. HCT116 cells were transfected with a GFP-tagged human TDP1 construct (GFP-TDP1^WT^). Cells were pre-treated with 1 μM aphidicolin (APH) for 15 min or 10 μM DRB for 1 h. Following CPT treatment (5 μM, 3 h), ectopic GFP-TDP1 was immunoprecipitated using anti-GFP antibody and the immune complexes were blotted with SDMA specific antibodies. The same blot was stripped and reprobed with anti-GFP antibody. (**B**) Densitometry analysis of arginine methylation of TDP1 (SDMA-TDP1) shown in panel A normalized against GFP TDP1. (**C**) PRMT5 depletion enhances replication-associated γH2AX. Confocal immunofluorescence microscopic analysis of CPT (5 μM, 3 h) induced γH2AX in control and PRMT5-depleted HCT116 cells pretreated with APH (1 μM, 15 min), DRB (10 μM, 1 h), or, both (APH +DRB, 1h) as indicated. PRMT5 and γH2AX are shown in red and green respectively. Nuclei were stained with DAPI (blue). (**D**) Quantification of replication and transcription associated CPT-induced γH2AX intensity per nucleus obtained from confocal immunofluorescence microscopy were calculated for 20–30 cells (calculated value ± S.E.M.) (h). APH induced reduction (fold change) in CPT-induced γH2AX intensity in PRMT5 proficient and PRMT5 depleted cells are indicated. Asterisks denote significant difference (**P < 0.001; *t* test) in CPT-induced γH2AX intensity between control and PRMT5 depleted cells.

Because Top1cc induce PRMT5-catalysed TDP1 arginine methylation (Figure 2), we examined the induction of TDP1 methylation upon Top1-mediated replication- and transcription-associated DNA breaks (2,43,44) by using the DNA polymerase inhibitor, aphidicolin (APH) to arrest replication, and 5,6-dichlorobenzimidazole 1-β-d-ribofuranoside (DRB) to inhibit transcription (10,43,44). Figure 4A and B shows that aphidicolin (APH) markedly suppressed CPT-induced TDP1 arginine methylation (SDMA), signifying that activation of TDP1 methylation is primarily replication-dependent. DRB partially abrogated CPT-induced TDP1-arginine methylation (Figure 4A and B), implying that transcription-induced damage by Top1cc has a more modest effect on TDP1-arginine methylation than replication damage.

To confirm the role of PRMT5 in response to Top1-mediated replication damage, we determined CPT-induced γH2AX foci in PRMT5-depleted cells in the presence of APH. Because APH arrests replication independently of PRMT5 expression (Supplementary Figure S4), CPT-induced γH2AX foci in APH-treated cells represent replication-independent Top1cc-induced DSBs (3,5,44). Figure 4C and D shows that APH markedly (∼4-fold) inhibited CPT-induced γH2AX foci in PRMT5-proficient cells, while APH had less effect (∼2-fold reduction) in PRMT5-deficient cells (Figure 4D). CPT-induced γH2AX in PRMT5-depleted cells was only partially abrogated by DRB (see quantification in Figure 4D). But combination of APH + DRB abrogated the CPT-induced γH2AX foci in PRMT5-depleted cells (Figure 4C and D), suggesting that the APH-resistant CPT-induced γH2AX foci are transcription-dependent. Taken together, our data indicate that PRMT5 activates TDP1 arginine methylation to repair Top1cc-induced replication- and transcription-mediated DSBs.

### Arginine methylation stimulates TDP1 catalytic activity

**Figure 5.**
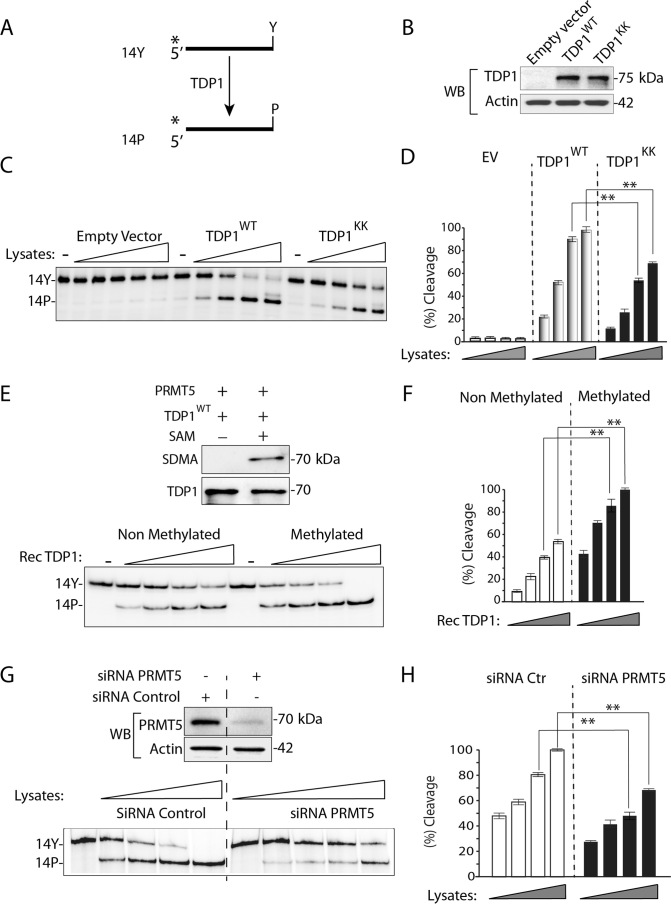
R361 and R586 methylation enhances the catalytic activity of TDP1. (**A**) Schematic representation of the TDP1 biochemical assays using a single-stranded oligopeptide 14Y. ^32^P-radiolabeling (*) was at the 5′-end of the oligopeptide. TDP1 catalyzes the hydrolysis of 3′-phosphotyrosine bond and converts the 14Y substrate to an oligonucleotide with 3′-phosphate, 14P. (**B**) Representative blot showing TDP1 levels in TDP1^−/−^ MEF cells expressing FLAG-TDP1^WT^, FLAG-TDP1^KK^, or empty vector. Actin served as loading control. (**C**) Representative gel autoradiographs showing TDP1 catalytic activity using cellular lysates from TDP1^−/−^ MEF cells expressing TDP1^WT^, TDP1^KK^, or empty vector (panel B). The cell lysates were normalized to yield similar protein concentrations (2 μg/μl) and serial dilutions (3-fold) were used to perform TDP1 activity assays. (**D**) Densitometry analysis of the gel shown in panel C. TDP1 mediated conversion of 14Y to 14P as a function of the concentration of serially diluted lysates as indicated). Error bars represent mean ± S.E. (*n* = 3). (**E**) TDP1 methylation stimulates its catalytic activity. Representative Western blot showing *in vitro* methylation levels of recombinant His-tagged wild-type TDP1 (TDP1) catalyzed by immunoprecipitated PRMT5 reacted with unlabeled S-adenosylmethionine (SAM). The same blot was stripped and reprobed with anti-TDP1 antibody showing the amount of substrate in each reaction. Representative gel showing TDP1 activity assays performed with non methylated vs. methylated TDP1. Indicated proteins after serial dilutions (3 fold) were used to perform TDP1 activity assays. (**F**) Densitometry analysis of non methylated vs. methylated TDP1 activity (panel E) as a function of serially diluted proteins as indicated. Error bars represent mean ± S.E. (*n* = 3). (**G**) siRNA knockdown of PRMT5 in HCT116 cells shows defective TDP1 activity. Western blots showing siRNA-mediated depletion of PRMT5 in HCT116 cells. Representative gel showing TDP1 activity assays using cell lysates from control and PRMT5 depleted cells. The cell lysates were normalized to yield similar protein concentrations (2 μg/μl). Serial dilutions (3-fold) were used for the TDP1 activity assays. (**H**) Densitometry analysis of TDP1 activity (panel G) as a function of concentration of serially diluted cell lysates as indicated. Error bars represent mean ± S.E. (*n* = 3).

To test whether PRMT5 has a functional impact on the 3′-phosphodiesterase activity of TDP1, we performed gel-based TDP1 activity assays (15,16,18,19,35,45). TDP1 catalyzes the hydrolysis of a 3′-tyrosyl-DNA nucleopeptide substrate (14-Y) to a product with a 3′-phosphate (14-P) with increased electrophoretic mobility (Figure 5A).

We employed an *ex vivo* approach with cellular extracts to test the impact of TDP1 arginine methylation on TDP1 catalytic activity (9,35). The advantage of employing cellular extracts is that the enzyme is maintained in its native structure and with its post-translational modifications. The assays were performed with cellular extracts from TDP1-knockout mouse embryonic fibroblasts (TDP1^−/−^) complemented either with wild-type TDP1 (FLAG-TDP1^WT^) or with the double arginine methylation mutant TDP1 (FLAG-TDP1^KK^). Both TDP1 constructs were expressed at similar levels (Figure 5B). Figure 5C shows that cellular extracts expressing methylation-deficient TDP1 (TDP1^KK^) were partially defective (∼2-fold) in converting the 14-Y substrate to the 14-P product compared to wild-type TDP1 (see quantification in Figure 5D). We also confirmed that FLAG- or GFP- tagged TDP1 showed similar catalytic activity under condition when both TDP1 constructs independent of their tag were expressed at similar levels (Supplementary Figure S1D - F). Recombinant enzymes (His-TDP1^WT^ and His-TDP1^KK^) exhibited similar levels of conversion of 14-Y to 14-P product (Supplementary Figure S2H). These experiments demonstrate that the TDP1^KK^ mutant is partially catalytically defective.

To demonstrate that arginine methylation stimulates the catalytic activity of TDP1, we conducted *in vitro* methylation of recombinant TDP1 with immunoprecipitated PRMT5 in the presence of S-adenosylmethionine (SAM) (Figure 5E, *top panel*), which was subjected to the gel based TDP1 activity assays (Figure 5E, bottom panel). Figure 5E and F shows that PRMT5-methylated TDP1 exhibits enhanced (∼3 fold) activity, demonstrating that TDP1 arginine methylation stimulates the catalytic activity of TDP1.

Next, we tested the impact of PRMT5 deficiency on TDP1 activity. Cellular extracts from PRMT5-deficient cells were employed for TDP1 activity assays. Figure 5G shows that cellular lysates from PRMT5-deficient cells were less active (∼3–4-fold) in TDP1 activity compared to the matched control (see quantification in Figure 5H). Collectively, these data demonstrate that PRMT5-mediated arginine methylation (SDMA) directly stimulates TDP1 catalytic activity.

### TDP1 arginine methylation promotes XRCC1 repair foci formation

**Figure 6.**
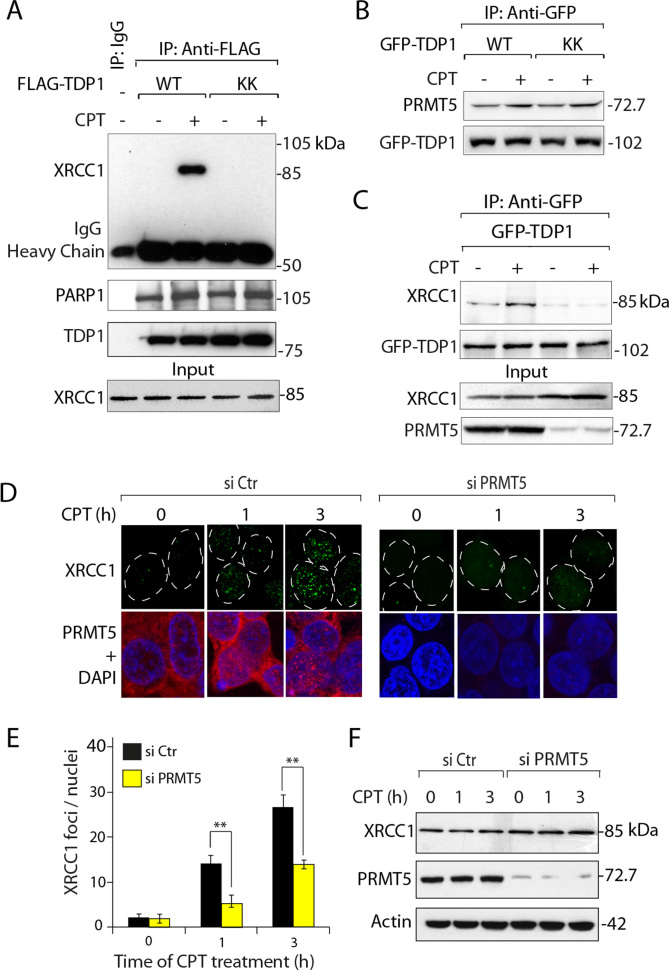
TDP1 methylation promotes TDP1-XRCC1 association and XRCC1 focal accumulation in response to Top1cc sites. (**A**) Wild-type (WT) and methylation-mutant (KK) flag-tagged TDP1 were ectopically expressed in HCT116 cells. After CPT treatment (5 μM, 3 h), ectopic TDP1 was immunoprecipitated using anti-flag antibody and the immune complexes were blotted with anti-XRCC1 antibody. The same blot was stripped and blotted with anti-PARP1 and anti-TDP1 antibody. Aliquots (10%) of the input show the similar level of XRCC1 prior to immunoprecipitation. Migration of protein molecular weight markers (kDa) is indicated at right. (**B**) Methylation mutant TDP1 (GFP-TDP1^KK^) was not deficient in interaction with PRMT5. HCT116 cells ectopically expressing GFP-TDP1 constructs (GFP-TDP1^WT^ and GFP-TDP1^KK^) were treated with or without CPT (5 μM, 3 h). GFP-TDP1 was immunoprecipitated using anti-GFP antibody and the immune complexes were blotted with anti-PRMT5 antibody. The same blot was stripped and reprobed with anti-GFP antibody to show equal loading. (**C**) PRMT5 depletion compromises the association of XRCC1 with TDP1. HCT116 cells were transfected with PRMT5 (siPRMT5) or control (Ctr) siRNA, followed by ectopic expression of GFP-TDP1^WT^. Following CPT treatment (5 μM, 3 h), ectopic GFP-TDP1 was immunoprecipitated using anti-GFP antibody and the immune complexes were blotted with anti-XRCC1 antibodies. The same blot was stripped and reprobed with anti-GFP antibody. Aliquots (10%) of the input show the level of PRMT5 knockdown, and XRCC1 prior to immunoprecipitation. Electrophoretic migration of protein molecular weight markers (kDa) is indicated at right. (**D**) Kinetics of appearance of nuclear XRCC1 foci in control and HCT116 cells transfected with PRMT5 siRNA. Cells were treated with CPT (5 μM). XRCC1 foci and PRMT5 are shown in green and red, respectively. Nuclei were stained with DAPI (blue). (**E**) Quantification of CPT-induced XRCC1 foci per nucleus (marked in dotted circle) obtained from immunofluorescence confocal microscopy were calculated for 20–25 cells (calculated value ± S.E.M.) and plotted as a function of time. Asterisks denote significant difference (**P < 0.001; *t* test) in CPT-induced XRCC1 foci between control and PRMT5 siRNA-transfected HCT116 cells. (**F**) siRNA knockdown of PRMT5 does not reduce XRCC1 expression. HCT116 cells were transfected with PRMT5 or control (Ctr) siRNA, then treated with CPT (5 μM) for indicated times (h) and protein levels (XRCC1 and PRMT5) were analyzed by Western blotting (Representative experiment is shown). Actin served as loading control. Electrophoretic migration of protein molecular weight markers (kDa) is indicated at right.

Because TDP1 is found in XRCC1 repair complexes (5,10,19,46), we tested the role of TDP1 arginine methylation on its association with XRCC1. First, co-immunoprecipitation (co-IP) of ectopic FLAG-TDP1^WT^ or FLAG-TDP1^KK^ showed that the TDP1^KK^ mutant was defective in pulling down XRCC1 after CPT treatment (Figure 6A), whereas the PARP1-TDP1 association (19) (Figure 6A) or TDP1-PRMT5 binding (Figure 6B) were similar with TDP1^WT^ and TDP1^KK^. Second, we tested TDP1-XRCC1 complex formation in PRMT5-depleted cells. Figure 6C shows that GFP-TDP1 was significantly defective in pulling down XRCC1 in PRMT5-depleted cells either in the presence or absence of CPT. Together, these results suggest that TDP1 methylation at R361 and R586 is critical for the association of TDP1 with XRCC1.

Next, we tested whether arginine methylation of TDP1 promotes XRCC1 foci formation (10,19). Immunofluorescence microscopy in untreated cells (Figure 6D) showed limited XRCC1 foci (Figure 6D). However, while PRMT5-proficient cells treated with CPT showed a time-dependent increase in nuclear XRCC1 foci, the PRMT5-depleted cells showed attenuated XRCC1 foci formation after CPT treatment (Figure 6E), and this effect was not due to reduced expression of XRCC1 (Figure 6F). We conclude that PRMT5-mediated arginine methylation is not only needed for TDP1-XRCC1 association but also for XRCC1 repair foci formation at Top1cc-induced DNA damage sites.

### Arginine methylation protects cells against DNA damage

**Figure 7.**
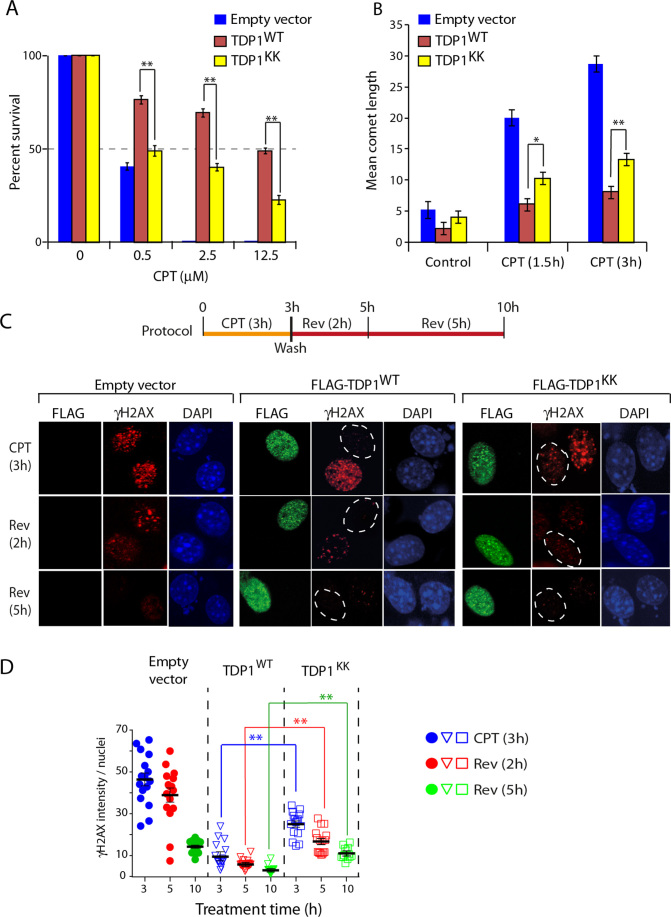
TDP1 arginine methylation at R361 and R586 protects cells against CPT-induced DNA damage. (**A**) Clonogenic survival of TDP1^−/−^ MEF cells expressing empty vector, TDP1^WT^ or TDP1^KK^ after treatment with the indicated concentrations of CPT for 3 h. Percent survival was normalized to the observed number of colonies from untreated control ± S.E.M. Asterisks denote statistically significant difference (**P < 0.001; *t* test). (**B**) Quantification of CPT-induced (5 μM) DNA strand breaks measured by alkaline comet assays in TDP1^−/−^ MEF cells expressing empty vector, FLAG-TDP1^WT^ or FLAG-TDP1^KK^ in a time-dependent manner as indicated. CPT**-**induced DNA strand breaks were calculated for 20–25 cells (mean ± S.E.M.). Asterisks denote statistically significant differences (**P < 0.001; *t* test). (**C**) γH2AX kinetics after CPT removal. TDP1^−/−^ MEF cells were transfected with FLAG-TDP1^WT^, FLAG-TDP1^KK^ or empty vector. Twenty four hours after transfection, cells were treated with CPT (5 μM, 3 h). After CPT removal (Rev), cells were cultured in drug-free medium for the indicated times (shown in top panel). Representative confocal images showing expression of FLAG-TDP1^WT^ or FLAG-TDP1^KK^ detected by immunofluorescence staining with anti-FLAG antibody (green). γH2AX induction is shown in red. Cells were counterstained with DAPI to visualize nuclei (blue). Nuclei are outlined in dashed white lines expressing ectopic FLAG-TDP1 variants. (**D**) Quantification of γH2AX intensity per nucleus after CPT removal obtained from immunofluorescence confocal microscopy were calculated for 20–25 cells (mean ± S.E.M.) and plotted as a function of time (h). Asterisks denote statistically significant difference (**P < 0.001; *t* test).

To establish the functional role of TDP1 arginine methylation *in vivo*, we tested whether expression of methylation-deficient flag-tagged human TDP1 (FLAG-TDP1^KK^) could rescue the CPT hypersensitivity of TDP1^−/−^ cells in survival assays (7,8,10). Figure 7A shows that transfection with FLAG-TDP1^KK^ failed to protect TDP1^−/−^ cells against CPT compared to transfection with FLAG-TDP1^WT^. Next, we measured DNA damage using alkaline comet assays (10). Figure 7B shows that TDP1^−/−^ cells expressing FLAG-TDP1^KK^ accumulated higher levels of CPT-induced DNA breaks than TDP1^−/−^ cells expressing FLAG-TDP1^WT^, which is consistent with the defective TDP1 activity of the methylation mutant TDP1 (see Figure 5).

Although Top1cc reverse within minutes after washing out CPT (3,47), DNA damage measured by γH2AX has much slower reversal kinetics (19). Therefore, we investigated the formation and disappearance of CPT-induced γH2AX using immunofluorescence microscopy. As expected, the levels of γH2AX in TDP1^−/−^ cells (complemented with vector control) were markedly higher than in TDP1^−/−^ cells complemented with wild-type TDP1 (FLAG-TDP1^WT^) (Figure 7C) (10). In contrast, cells transfected with FLAG-TDP1^KK^ showed significantly higher γH2AX compared to their wild-type counterpart (Figure 7C). After washing out CPT, TDP1^−/−^ cell expressing FLAG-TDP1^KK^ showed more persistent γH2AX foci (Figure 7C and D). These results demonstrate that expression of a non-methylable allele of TDP1 results in defective repair.

## DISCUSSION

**Figure 8.**
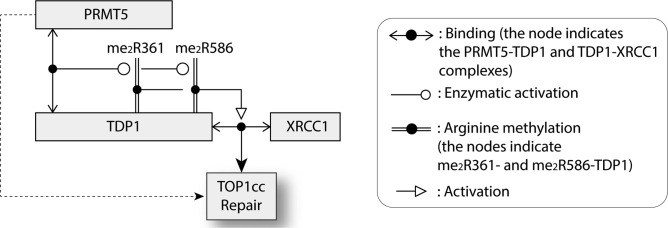
Schematic representation of the activation of TDP1 by its arginine methylation at R361 and R586 by PRMT5. Symbols are indicated at right and details are provided in the Discussion.

The present study reveals that arginine methylation of TDP1 is a major regulatory factor for TDP1-mediated DNA repair. Figure 8 summarizes our findings demonstrating the direct binding of PRMT5 to TDP1 and showing that PRMT5 catalyzes TDP1 methylation at residues R361 and R586 both *in vitro* and *in vivo*. We establish that arginine methylation promotes TDP1 catalytic activity and its association with XRCC1, thereby facilitating the formation of XRCC1 repair foci. Enhanced formation of Top1-associated DSBs in cells lacking TDP1 or expressing the non-methylated TDP1 (TDP1^KK^) imply a previously unknown role of PRMT5 in the repair of DNA damage induced by Top1 inhibitors.

Post-translational modifications (PTM) play key roles in ensuring efficient propagation of damage signals for DNA repair (23,24). While arginine methylation is an established key epigenetic mark regulating gene expression and cell proliferation, its emerging role in coordinating the optimal activity of non-histone proteins in the DNA damage response pathways (DDR) demonstrates that arginine methylation is akin to other PTM involved in the DNA damage response (DDR) (26–29,48,49). Hence, TDP1 can now be added to the DDR substrates of PRMT5.

PRMT5, the major arginine methyltransferase catalyzing SDMA modifications (26) is commonly activated in cancers (26,27). Genetic inactivation of PRMT5 in mice is early embryonic lethal (50), while PRMT5 depletion causes cell proliferation defects (51). The role of PRMT5 in Top1cc repair can be derived from our data showing that PRMT5 knockdown cells have defective TDP1 activity (Figure 5G and H) and elevated CPT-induced DSBs, ADP-ribose polymers and lethality (Figure 3). Consistent with the role of PRMT5 in DDR signaling, deficiency in PRMT5 reduces p53 levels leading to cell cycle checkpoint defects and cell death (34). Rad9, another PRMT5 substrate, is regulated by PRMT5 for replication damage checkpoint activation and resistance to hydroxyurea-induced DNA damage (33). The replication and repair endonuclease FEN1 is also controlled by PRMT5. Deficiency in FEN1 symmetric arginine methylation (SDMA) by PRMT5 has been implicated in defective long-patch base excision repair (BER), replication delay and genomic instability (31). When a replication fork proceeds toward a stalled Top1cc, the extension of the leading strand is terminated with replication fork run-off, resulting in a Top1-linked double-stranded end (2,3,52). Here we show that Top1-induced replication damage induces TDP1 arginine methylation (Figure 4A), which is consistent with the role of PRMT5 in the repair of replication-associated Top1cc (Figure 4). Our findings unveil a novel physical and functional association between PRMT5 and TDP1 ensuring the repair of Top1cc-associated DNA damage and genome maintenance. TDP1 and PRMT5 are plausibly working in additional pathway as revealed by the additional sensitivity to CPT upon double inactivation of TDP1 plus PRMT5 (Figure 3C). Our results imply that the enhanced camptothecin sensitivity is not solely mediated by the failure to arginine-methylate TDP1 (and dotted arrow in Figure 8), and is in keeping with the fact that PRMT5 is involved in several DDR and replication response pathways including transcriptional regulation, RNA metabolism ribosome biogenesis, Golgi apparatus structure maintenance, epigenetic regulation, DNA repair pathways and genome maintenance (26–28,42). Nonetheless, our results reveal that PRMT5 depletion reduces TDP1 catalytic activity, abrogates repair complex formation with XRCC1 and CPT-induced Top1cc repair.

The known PTM regulation sites for TDP1 primarily involve its N-terminal domain (NTD; see Figure 2A), which is dispensable for TDP1 catalytic activity (5,10). Prior studies showed that the N-terminal region of TDP1 is required for the formation of PARP1-TDP1 complexes and that PARylation is required for the detection and repair of Top1cc in the context of XRCC1 repair complexes (19). Two other NTD post-translational modifications (PTM) of TDP1 regulate its activity. TDP1 phosphorylation by ATM and DNA-PK at serine 81 enhances TDP1 activity by promoting its stability and interaction with ligase III (10,22). TDP1 SUMOylation at lysine 111 also promotes DNA repair and recruitment of TDP1 to DNA damage sites (25). Our study shows that the N-terminal domain of TDP1 (1-185 amino acids) also binds the N-terminal domain (1-293 amino acids) of PRMT5 (Figure 1 and Scheme in Figure 8). Consistently, the N-terminus of PRMT5 harbors a TIM-barrel domain that functions as a structural scaffold for recruiting PRMT5 substrates and association with MEP50 (40), which is in agreement with the fact that N-terminal deletion of PRMT5 inhibits its interaction with TDP1 (Figure 1E). In turn, PRMT5 catalyzes TDP1 methylation at R361 and R586, two residues in the catalytic core of TDP1 (see Figure 2 and Supplementary Figure S2, and Figure 8). Crystal structure analyses (53) show that both R361 and R586 are on the surface of TDP1 outside the catalytic HKN motifs of TDP1 (see Figure 2A), which is consistent with their accessibility for SDMA modification by PRMT5, and with the fact the double mutant TDP1^R361K, R586K^ remains catalytically active (see Figure 5). Yet, our data further establish the stimulation of TDP1 activity by PRMT5 using *in vitro* methylation of recombinant TDP1 (Figure 5E and F). Consistent with these data, extracts from PRMT5-depleted cells demonstrate reduced TDP1 catalytic activity (Figure 5G and H).

Our findings extend the role of the arginine methyltransferases in DNA repair and DNA damage responses (26,28). PRMT5 acts directly by catalyzing methylation of TDP1 (present report), FEN1, RAD9 and p53 (31,33,34). PRMT1 and PRMT6, the methyltransferases responsible for asymmetric dimethylation of arginine residues also enhance DNA repair by methylating MRE11, 53BP1, BRCA1 and DNA polymerase β (26,28,48,49). Hence, both PRMT5 and PRMT6 are now involved in the repair of Top1cc. Polymerase β is a component of the base excision repair which form complexes with TDP1 (2,54). As TDP1 generates 3′-phosphate DNA termini, PNKP converts the 3′-phosphate to 3′-hydroxyl end before they can be extended by polymerase β and further sealed by XRCC1-ligase III (5,13,54). As XRCC1 is implicated in Top1cc repair (5,10,19,55), it is notable that defective TDP1 activity in PRMT5 knockdown cells affects the recruitment of XRCC1 repair foci at Top1cc-induced DNA damage sites (Figure 6D). Lastly, activation of the exonuclease activity of MRE11 by PRMT1 (28,49) is also likely to contribute to Top1cc repair as Mre11, which is a part of the MRN complex (Mre11/Rad50/Nbs1) constitutes an alternative pathway for the excision of Top1-DNA adducts (2,5,6).

In conclusion, the present study reveals the significance of PRMT5 for the repair of Top1cc. It also suggests the importance of PRMT5 as a potential resistance determinant to clinically used camptothecins derivatives (topotecan and irinotecan), and as a potential target for combination therapy with these Top1 inhibitors. Further studies are warranted to determine the potential relevance of PRMT5 for the other DNA repair functions of TDP1 (5). Impending evidence also suggest a role of PRMT5 in tumorigenesis including leukemia, lymphoma, and in many solid tumors, making PRMT5 an attractive anticancer target (26,51,56–58).

## Supplementary Material

Supplementary Data
