# Supplementary material for: PRMT5-mediated arginine methylation of TDP1 for the repair of topoisomerase I covalent complexes
Source: Nucleic Acids Res. 2018 Apr 30;46(11):5601–17. doi: 10.1093/nar/gky291 (PMC6009676; doi:10.1093/nar/gky291)
Supplement: Supplementary Data [file gky291_supplemental_figures.pdf]

SUPPLEMENTARY DATA

PRMT5-mediated arginine methylation of TDP1 for the repair of topoisomerase I covalent complexes

Ishita Rehman[1], Suparna M Basu[1], Subhendu K Das[1], Sangheeta Bhattacharjee [1], Arijit Ghosh[1], Yves Pommier [2,*], and Benu Brata Das [1,2*]

[1]Laboratory of Molecular Biology, Department of Physical Chemistry, Indian Association for the Cultivation of Science, Jadavpur, Kolkata-700032, INDIA. [2]Developmental Therapeutics Branch and Laboratory of Molecular Pharmacology, Center for Cancer Research, National Cancer Institute, National Institutes of Health, Bethesda, MD 20892-4255, USA.

Supplementary Figure:

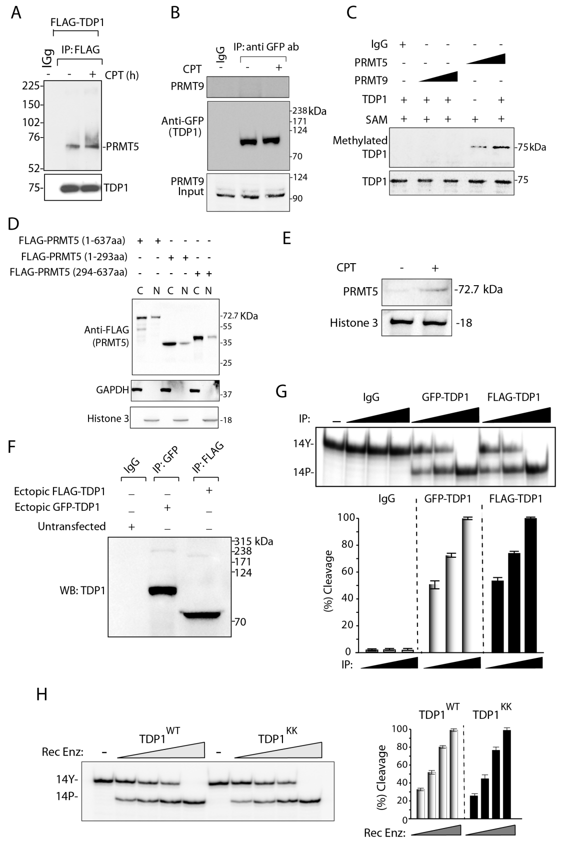

Supplementary Figure S1: (A) TDP1-PRMT5 association is independent of the TDP1 fusion tag. HCT116 cells ectopically expressing FLAG-TDP1 treated with or without CPT (5 μM, 3 h), were immunoprecipitated using anti-flag antibody and the immune complexes were blotted with anti-PRMT5 antibody. The same blot was stripped and reprobed with anti-TDP1 antibody. Migration of protein molecular weight markers is indicated at left. **(B)** Same as **(A)** except HCT116 cells ectopically expressing GFP-TDP1 in the presence or absence of CPT (5 μM, 3 h),

were immunoprecipitated using anti-GFP antibody and the immune complexes were blotted with anti-PRMT9 antibody. The same blot was stripped and reprobed with anti-GFP antibody to show the expression of the GFP-TDP1. Aliquots (10 %) of the input show the level of PRMT9 prior to immunoprecipitation. Migration of protein molecular weight markers (kDa) is indicated at right. **(C)** *In vitro* methylation assay with PRMT9 or PRMT5 immunoprecipitated from HCT116 cells with unlabeled S-adenosylmethionine (SAM). The substrate was recombinant His-tagged TDP1. The immune complexes were normalized to yield similar protein concentrations (2 µg ⁄ µl) and serial dilutions (3-fold) were used to perform *in vitro* methylation assays. The same blot was stripped and reprobed with anti-TDP1 antibody showing the amount of substrate in each reaction. **(D)** PRMT5 is predominantly localized in the cytoplasm. Representative Western blots showing the cellular localizations of flag-tagged human PRMT5 (1-637 aa), truncated N-terminal domain (1-293 aa) and truncated C-terminal domain (294-637 aa). Flag-tagged PRMT5 constructs were ectopically expressed in HCT116 cells and cytoplasmic and nuclear fractions were isolated and probed with anti-flag antibodies. The fractions were also probed with GAPDH and Histone 3 to demonstrate the purity of cytoplasmic (C) and nuclear (N) preparations respectively. Migration of protein molecular weight markers (kDa) is indicated at right. **(E)** CPT accumulates PRMT5 in the chromatin. Representative Western blots showing the chromatin bound levels of PRMT5. HCT116 cells were treated with or without CPT (5 µM, 3 h) and chromatin bound fractions were isolated and probed with anti-PRMT5 antibody. Histone 3 served as loading control **(F)** GFP-tagged TDP1, or, Flag-tagged TDP1, were ectopically expressed in TDP1-/- mouse fibroblasts cells and were immunoprecipitated using anti-GFP, or, anti-flag antibody, respectively, as indicated. The immune complexes were blotted with anti-TDP1 antibody to show similar levels of TDP1 pull-down. **(G)** Representative gel showing TDP1 activity assays performed with purified immune complexes of GFP-TDP1 and FLAG-TDP1 as source of TDP1 or with anti-IgG antibody. The GFP and FLAG-immune complexes were normalized to yield similar protein concentrations (0.5 µg ⁄ µl) and serial dilutions (3-fold) were used to perform TDP1 activity assays**.** Densitometry analysis of TDP1 activity (top panel) as a function of serially diluted immune complexes as indicated. Error bars represent mean ± S.E. (n=3). **(H)** Representative gel autoradiographs showing TDP1 catalytic activity using recombinant $TDP1^{WT}$ or $TDP1^{KK}$.The recombinant proteins were normalized to yield similar protein concentrations (0.5 µg ⁄ µl) and serial dilutions (3-fold) were used to perform TDP1 activity assays. Densitometry analysis of the gel shown in left panel. TDP1 mediated conversion of 14Y to 14P as a function of the concentration of serially diluted recombinant proteins as indicated. Error bars represent mean ± S.E. (n=3).

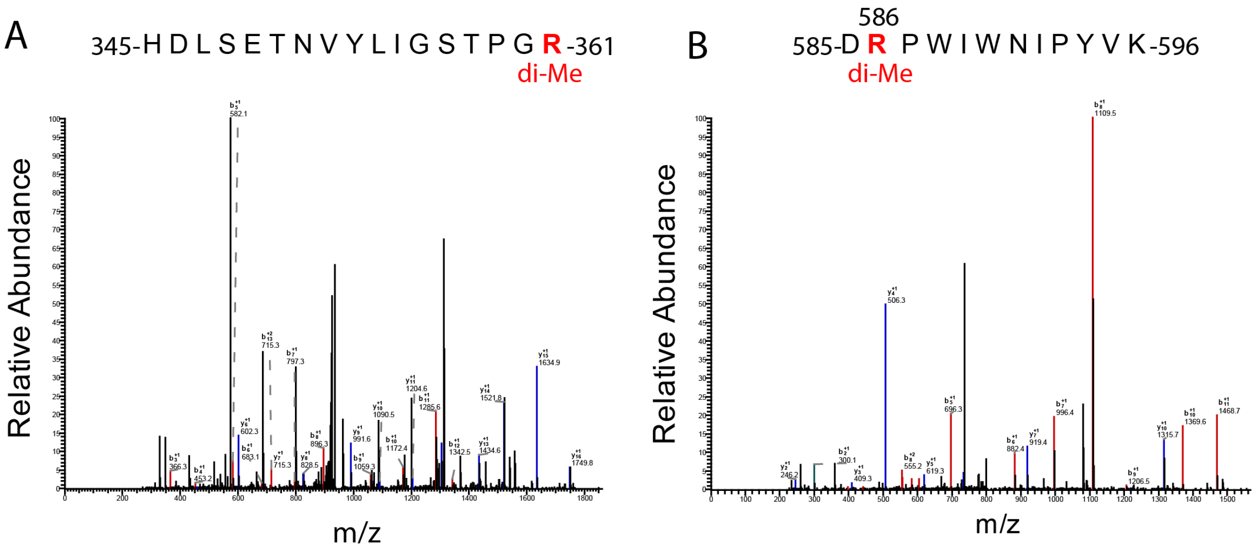

Supplementary Figure S2: Mass spectrometry profile of TDP1 as detected by LC-MS/MS. The sequence and the mass spectrometry profile of TDP1 covering residues **(A)** 345–361 aa and **(B)** 585-596 aa are shown, and the dimethylated arginine side chains are indicated in red. Note: TDP1-R361 dimethylation was detected in cells ectopically expressing FLAG-TDP1 after CPT treatment, while TDP1-R586 dimethylation was detected independent of DNA damage.

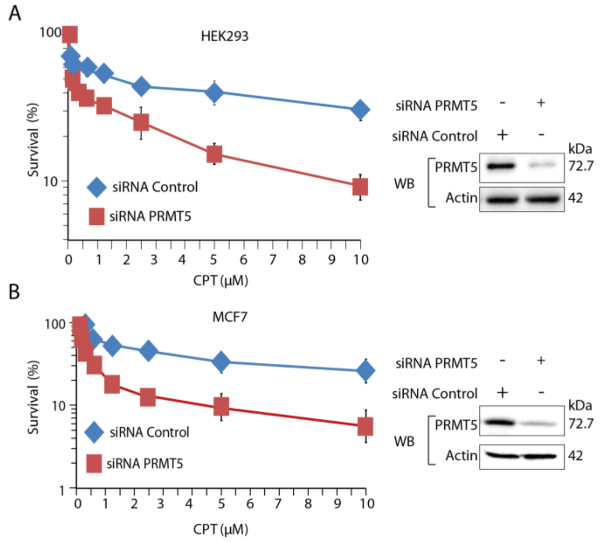

Supplementary Figure S3: PRMT5 knockdown sensitizes different cells to CPT. Cell survival curves of HEK293 **(A) and** MCF 7 **(B) cells,** transfected with PRMT5 or control siRNA. Western blots showing siRNA-mediated depletion of PRMT5. CPT-induced cytotoxicity (%) was calculated with respect to the untreated control. Each point corresponds to the mean ± S.D. of at least three experiments. Error bars represent SD (n=3).

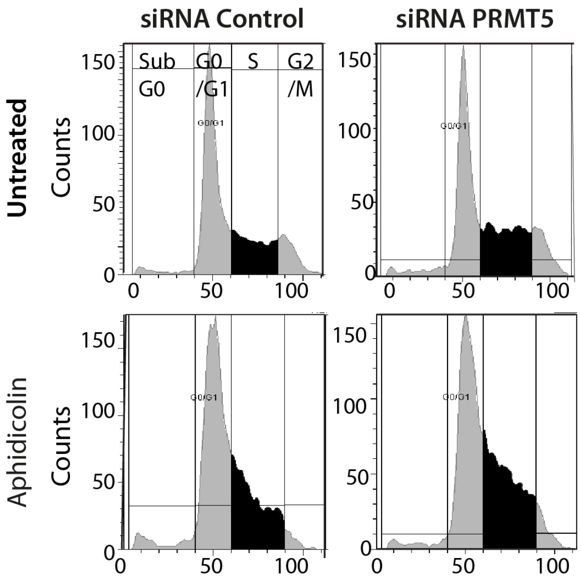

Sample / Phase	siRNA Ctrl	siRNA PRMT5	siRNA Ctrl + APH	siRNA PRMT5 + APH
G0/G1	53.9%	47.4%	57.4%	50.4%
**S**	**28.2%**	**33.1%**	**34.5%**	**40.0%**
G2/M	11%	13.4%	2.9%	4.9%

Supplementary Figure S4: PRMT5 depletion primarily induces replication damage. PRMT5 depletion promotes S-phase arrest. Following transfection with PRMT5 or control (Ctrl) siRNA for 48 h, HEK293 cells were treated with or without APH (5 µg / ml, 24 h), as indicated. Cells were then harvested, stained with propidium iodide and analyzed by FACS. Percentages of cells in the G0/G1, S (indicated in black), or, G2/M phases of the cell cycle as determined from the FACS histograms shown in the left panel are illustrated on the table (right panel).
